# Clinical efficacy study on the efficacy and safety of acupuncture for post-stroke dysphagia based on the regulation of the swallowing neural circuit: protocol for a multicenter, open-label, randomized controlled trial

**DOI:** 10.3389/fmed.2025.1697235

**Published:** 2025-12-10

**Authors:** Yuhua Yan, Guoshan Zhang, Nan Li, Jian Luo, Bo Li, Lian Liu, Cai Li, Zenghui Yue, Murong Zhimiao

**Affiliations:** 1 School of Acupuncture, Moxibustion, Tuina and Rehabilitation, Hunan University of Chinese Medicine, Hunan, China; 2 First Affiliated Hospital of Hunan University of Chinese Medicine, Hunan, China; 3 Second Affiliated Hospital of Hunan University of Chinese Medicine, Hunan, China

**Keywords:** dysphagia, stroke, acupuncture, fMRI, sEMG, study protocol

## Abstract

**Introduction:**

Post-stroke dysphagia (PSD) is a common complication of stroke. Rehabilitation training and acupuncture have been suggested as current effective therapies, but high-quality evidence of acupuncture is insufficient. A meta-analysis has shown that acupuncture may be an effective adjunctive treatment for improving swallowing function, but the guidelines do not provide sufficient recommendations. Therefore, clinical and mechanism-related research on acupuncture treatment for post-stroke dysphagia should be vigorously promoted.

**Methods:**

This study is a multicenter, open-label, randomized clinical trial. A total of 115 participants will be enrolled: 100 PSD patients will be randomly allocated in a 1:1 ratio to the experimental group (acupuncture combined with basic treatment) or the control group (rehabilitation training combined with basic treatment), and 15 healthy controls will only provide baseline information without receiving any treatment. Both groups will be given a 4-week intervention (three times per week). The primary efficacy outcome measure is the Functional Oral Intake Scale (FOIS). Secondary outcome measures include: Regional Homogeneity (ReHo) and Amplitude of Low-Frequency Fluctuation (ALFF) metrics from Functional Magnetic Resonance Imaging (fMRI) scans, surface electromyography (sEMG) assessments, the Swallowing Disorders-Specific Quality of Life Questionnaire (SWAL-QOL), the National Institutes of Health Stroke Scale (NIHSS), the Treatment Expectation Questionnaire (TEX-Q) and patient-reported outcomes (PRO). Additionally, the Fiberoptic Endoscopic Evaluation of Swallowing (FEES) will be performed together with the Penetration-Aspiration Scale (PAS) and the Yale Pharyngeal Residue Severity Rating Scale (YPR-SRS). Outcomes will be assessed at five time points: Baseline, day 15 and day 30 during treatment, and 30 and 60 days after treatment. All primary analyses will be conducted using both intention-to-treat and per-protocol approaches.

**Discussion:**

This trial will investigate the efficacy of acupuncture targeting swallowing neural circuit regulation for improving swallowing function, reducing major complications, and enhancing quality of life in patients with swallowing disorders. It will also explore the underlying neural regulatory mechanisms, evaluate the efficacy of acupuncture for post-stroke dysphagia, and provide high-quality evidence.

**Clinical trial registration:**

https://itmctr.ccebtcm.org.cn/mgt/project/user/project-view/F5B9D5CA-0B85-47BD-8911-5B2B0DDD4EB5, identifier ITMCT R2025001435.

## 1 Introduction

In 2020, the incidence of stroke among adults in China was reported as 310.0 cases per 100,000 person-years (1), and epidemiological projections suggest a sustained increase from 2019 to 2050 (2). Currently, the incidence of post-stroke dysphagia ranges from 37 to 78% (3). Dysphagia can lead to dehydration, malnutrition, aspiration pneumonia, and other complications (4), and may also exacerbate post-stroke mortality and disability (5).

The main treatments for post-stroke dysphagia (PSD) include behavioral interventions, rehabilitative training, pharmacotherapy, and neuromuscular electrical stimulation (NMES) (6). The 2021 AHA/ASA Guidelines for Adult Stroke Rehabilitation (originally published in 2016) suggest that acupuncture may be considered as an adjunctive therapy for dysphagia (Class IIb recommendation, Level B evidence). Clinical studies (7–9) increasingly support the therapeutic benefits of acupuncture in improving swallowing function after stroke (10). Neurophysiological research (11) has clarified the modulatory pathways of the swallowing neural circuit, indicating that acupuncture may act through targeted modulation of this network. These findings provide a mechanistic basis for further research on acupuncture in pathological conditions.

This study aims to evaluate acupuncture’s therapeutic efficacy for post-stroke dysphagia (PSD) by rigorously comparing it with conventional swallowing rehabilitation. Although previous research has demonstrated acupuncture’s potential benefits, direct comparisons with established rehabilitation methods remain lacking. Moreover, the neural mechanisms underlying acupuncture’s effects on PSD require further clarification. Given the substantial differences between acupuncture and rehabilitation procedures in clinical trials, implementing a single-blind design is challenging. Therefore, an open-label, multi-center, randomized controlled trial will be adopted to comprehensively evaluate the efficacy of acupuncture for PSD patients, clarify the neural mechanisms involved in acupuncture intervention for post-stroke dysphagia and obtain high-quality evidence-based data.

## 2 Methods and analysis

### 2.1 Objective

This study aims to explore acupuncture’s efficacy in post-stroke dysphagia (PSD) patients, clarify the neural mechanisms of acupuncture intervention for PSD, and obtain high-quality evidence.

### 2.2 Study design

This is a multicenter, open-label, randomized controlled trial. A total of 115 participants will be enrolled, including 100 PSD patients to be randomly allocated in a 1:1 ratio to either the experimental group or the rehabilitation control group, and 15 additional participants will serve as the healthy control group. The study period will include baseline observations and 4 weeks of treatment. The clinical efficacy of acupuncture intervention will be assessed using functional assessment scales on day 15, day 30 during treatment, and at 30 and 60-day follow-ups after treatment completion.

**FIGURE 1:**
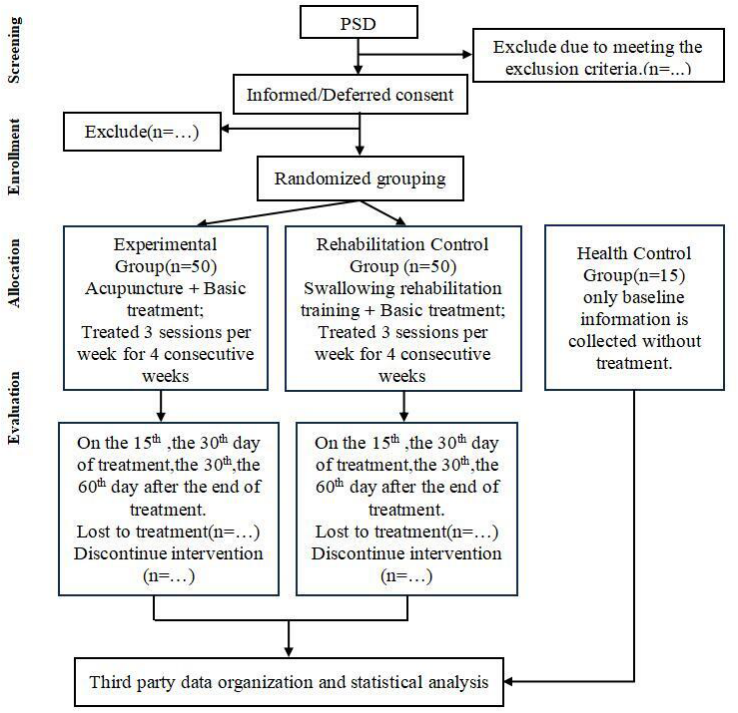
Trail flowchart.

This clinical trial adheres to the Consolidated Standards of Reporting Trials (CONSORT) guidelines (12) and complies with the Standards for Reporting Interventions in Clinical Trials of Acupuncture (STRICTA) (13) to ensure methodological rigor in acupuncture-specific interventions. The study design flowchart is presented in Figure 1.

### 2.3 Inclusion criteria

Patients are eligible for study inclusion if they meet all of the following:

(1)    Fulfill the diagnostic criteria for acute ischemic stroke as defined by the Chinese Guidelines for the Diagnosis and Treatment of Acute Ischemic Stroke (2023 Edition) (14), and have dysphagia confirmed by the Scottish Intercollegiate Guidelines Network (SIGN) recommendations (15).
(2)    Age between 18 and 80 years.
(3)    FOIS levels between 2 and 4.
(4)    Stroke duration of 15–180 days.
(5)    Infarction location in the cerebral cortex and subcortical regions.
(6)    Patients or their legal guardians must fully understand the study procedures, provide written informed consent, and agree to participate.

### 2.4 Exclusion criteria

Patients will be excluded if they have any one of the following:

(1)    Pre-existing dysphagia unrelated to the current stroke.
(2)    Life-threatening comorbidities with < 3-month life expectancy.
(3)    Severe psychiatric/cognitive impairments affecting study compliance.
(4)    Unable to tolerate acupuncture.
(5)    Inability to complete standardized swallowing assessments.
(6)    Current pregnancy or lactation.
(7)    Participated in other clinical trials within 1 month.

### 2.5 Dropout criteria

(1)    Subjects who have poor compliance during the clinical trial, or are unwilling to continue participating in the study and withdraw voluntarily.
(2)    Those who experience serious adverse reactions, serious complications, or deterioration during the study are unfit to continue.
(3)    Subjects who did not follow the treatment protocol or had incomplete observation data that affected the assessment.

### 2.6 Recruitment of study participants

The study will be conducted at two hospitals of Hunan University of Traditional Chinese Medicine (First and Second Affiliated Hospitals), with the Second Affiliated Hospital leading study design and implementation. PSD participants will be recruited from hospital wards/clinics (April 2024-June 2025), while healthy controls will be enrolled from the community during the same period.

### 2.7 Sample size, randomization, and blinding

The primary efficacy outcome is the FOIS score. As neither our team nor previous studies have used this measure to assess acupuncture efficacy for post-stroke dysphagia, it is not possible to determine an appropriate effect size from existing data. A review of similar studies has revealed that after 4 weeks, the FOIS score difference from baseline was 1.31 (rehabilitation training group) vs. 0.31 (sham training group), with a standard deviation of 1.09 (16). This value will be used as the expected effect size for this trial, and sample size calculations will be conducted based on a superiority trial design (17). These parameters (μA = 1.31, μB = 0.31, σ = 1.09, α = 0.05, power = 0.8, δ = 0.39) were applied in a superiority design with 1:1 allocation. After calculation, nA = nB = 40. Considering a 20% dropout rate, this study is expected to enroll 100 patients.

Randomization will use computer-generated numbers to allocate patients equally to acupuncture or rehabilitation groups. A stratified block randomization design will be employed. An independent statistician will generate all random sequences in SPSS 25.0, with study center and baseline FOIS as stratification factors, block lengths of 4 and 6 randomly interleaved, and the seed number set to 20240420. Allocation concealment will be implemented through sequentially numbered, opaque, sealed envelopes kept by a research coordinator who has no conflicts of interest with the study team. After participants have completed baseline assessments and provided written informed consent, the responsible physician will open the corresponding envelope on-site to reveal the allocation. The statistician generating the random sequence will operate independently of the coordinator handling allocation concealment. Neither patients nor physicians will know the allocation in advance. All participants will be followed for 90 days.

### 2.8 Intervention

The intervention protocols are designed based on traditional Chinese medicine theory (TCM), clinical guidelines, and expert consensus. All acupuncturists must hold valid Chinese medical practitioner license and has completed specialized training. All participants will receive standard treatment for ischemic stroke, including blood pressure control, blood glucose control, lipid control, antiplatelet therapy, and other symptomatic and preventive treatments. Specific treatment recommendations are based on the guidelines outlined in the Chinese Guidelines for the Diagnosis and Treatment of Acute Ischemic Stroke (2023) (14) and the Chinese Guidelines for Secondary Prevention of Ischemic Stroke and Transient Ischemic Attack (2022) (18). Specific medications, dosages, and target goals are detailed in Table 1. Swallowing rehabilitation training will be applied three sessions per week under professional supervision for four consecutive weeks.

**TABLE 1:** Standardized secondary prevention protocol for ischemic stroke.

Parameter	First-line drug and dose	Target value	Monitoring time-point	Non-adherence management
Blood pressure	Amlodipine 5 mg once daily or irbesartan 150 mg once daily; add the alternate agent if BP uncontrolled after 2 weeks	< 130/80 mmHg	Baseline, 2, 4, 8 weeks	If BP remains ≥ 140/90 mmHg at two consecutive visits, investigators will uptitrate the dose or initiate dual therapy; if target is still not achieved, the case will be recorded as a protocol deviation.
LDL-cholesterol	Atorvastatin 20 mg once nightly	< 1.8 mmol/L or a ≥ 50 % reduction from baseline	Baseline, 4, 8 weeks	If LDL-C remains ≥ mmol/L, add ezetimibe 10 mg once daily and review diet adherence
Glycaemic control	Metformin 0.5 g twice daily; step-wise insulin regimen if intolerance or HbA1c > 10 %	Fasting plasma glucose 4.4–7.0 mmol/L; HbA1c < 7 %	Weekly finger-stick; venous sample every 4 weeks	If two consecutive fasting glucose values exceed 10 mmol/L, investigators will initiate basal-bolus insulin.
Antiplatelet therapy	Aspirin 100 mg once daily; if aspirin-intolerant, clopidogrel 75 mg once daily	Patients will continue the assigned antiplatelet dose.	Baseline, every follow-up	Missed doses ≥ 3 consecutive days = non-adherence; counsel and re-educate; switch drug if adverse event
Swallowing basic care	Oral hygiene, thickened fluid, postural adjustment per Scottish Intercollegiate Guidelines; no ad libitum oral intake except study diet	Daily actual intake recorded	Daily by study nurse	Any unauthorized oral intake recorded as protocol deviation

#### 2.8.1 The experimental group

**FIGURE 2:**
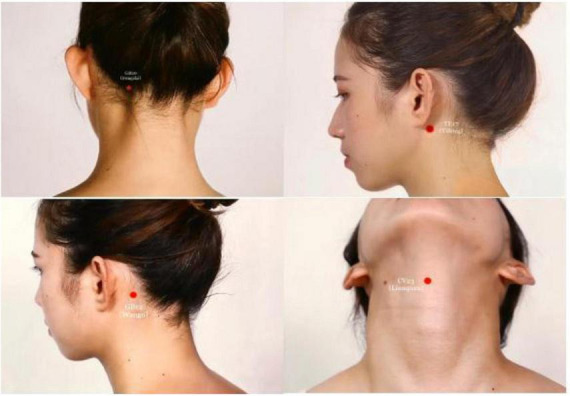
Location of acupuncture points (Source: Atlas of meridians and acupuncture points by JiuDaifu).

Disposable sterile acupuncture needles (0.30 mm × 40 mm and 0.30 mm × 75 mm, Hwato brand, Suzhou Medical Supplies Factory Co., Ltd., China) will be used. The “Tong Guan Li Qiao” acupuncture therapy (19) will be administered according to TCM theory. The protocol includes the following bilateral acupoints: GB20 (Fengchi), GB12 (Wangu), TE17 (Yifeng), and CV23 (Lianquan) (Figure 2 and Table 2).

**TABLE 2:** Anatomical location of selected acupoints.

Acupoints	Location
GB20 (Fengchi)	In the posterior region of the neck, under the occiput, in the depression between the upper part of the sternocleidomastoid muscle and the upper part of the trapezius muscle.
GB12 (Wangu)	On the neck, at the posterior and inferior depression of the mastoid process behind the ear.
TE17 (Yifeng)	On the neck, behind the earlobe, at the anterior and inferior depression of the mastoid process.
CV23 (Lianquan)	In the anterior region of the neck, on the anterior median line, above the laryngeal prominence, and in the depression of the upper edge of the hyoid bone.

Patients will lie in a supine position. GB20 (Fengchi) needles will be bilaterally inserted at a 30° inferior angle toward the thyroid cartilage to a depth of 2.5–3.0 cun (≈65–75 mm). Small-amplitude, high-frequency twisting manipulation will be applied for 1 min to achieve strong stimulation, targeting a throat numbness/swelling sensation called “deqi.” GB12 (Wangu) and TE17 (Yifeng) will be needled with the same depth, angle, and technique as GB20. CV23 (Lianquan) will be inserted to 1.0–1.5 cun (≈25–40 mm) with lifting-thrusting manipulation. All needles will be retained for 30 min. Participants will receive three sessions per week for four consecutive weeks.

Deqi will be assessed subjectively using the Chinese version of the Massachusetts General Hospital Acupuncture Sensation Scale (c-MASS) (20), which evaluates 12 distinct sensations to quantify deqi intensity and quality. Participants will self-complete the c-MASS within 30 s after each needle manipulation. In accordance with prior domestic research (21), “adequate deqi” will be defined as a total c-MASS score ≥ 4, accompanied by at least two typical sensations (soreness, numbness, fullness, or heaviness) each rated ≥ 3. Cases not fulfilling both criteria will be classified as “inadequate deqi.” The Deqi Quality Index (DQI) for each participant will be calculated as the mean of the total c-MASS scores recorded at three time points: initiation of intervention, 15 and 30 min.

During participant completion of the c-MASS, acupuncturists will simultaneously record their “hand sensation” using a visual analog scale (VAS). All acupuncturists will receive standardized training before the study begins, including consistent anchor definitions for the 0- and 10-point marks on the VAS, to minimize inter-practitioner scale drift. All deqi-related data will be documented in case report forms (CRF).

#### 2.8.2 The rehabilitation control group

The rehabilitation control group will receive swallowing rehabilitation training three sessions per week under the guidance of professionals, with specific operations as follows:

##### 2.8.2.1 Oral motor training

(1)    Patients perform active lip and tongue movement exercises under guidance.
(2)    Tongue pressure resistance training: A biofeedback device is used to improve tongue pressure and mobility.
(3)    Tongue muscle rehabilitation: Use a tongue muscle rehabilitation trainer (suction device) to apply passive traction or active-assistive movement and resistance training during tongue movement.
(4)    Masako maneuver: The tongue is held in protrusion during swallowing to facilitate anterior movement of the pharyngeal wall, increasing pharyngeal pressure and accelerating the progression of the bolus.
(5)    Shaker exercise: In supine position, patients elevate the head (shoulders remain on bed) to direct their gaze toward their toes. While holding the head at the highest point, they will maintain this position for 60s × 3 repetitions (with 60s rest intervals); then maintain a steady pace to raise the head and visualize toes 30 times.

##### 2.8.2.2 Airway protection methods

(1)    Mendelsohn maneuver: Patients are trained to voluntarily prolong laryngeal elevation and maintain elevation for ≥ 1.5 s per swallow.
(2)    Supraglottic Swallowing: Patients are trained to close the glottis before swallowing initiation to prevent aspiration of food or liquids, and to cough immediately after swallowing to clear any remaining food from the vocal cords.
(3)    Effortful swallow: Patients are trained to perform multiple forceful swallows during the pharyngeal phase of swallowing.

#### 2.8.3 The healthy control group

At baseline, functional MRI will be conducted to assess Regional Homogeneity (ReHo) and Amplitude of Low-Frequency Fluctuations (ALFF), together with Fiberoptic Endoscopic Evaluation of Swallowing (FEES). The study will not involve additional interventions or follow-up assessments, as published evidence (22, 23) supports the 12-month stability (ICC ≥ 0.75) of these resting-state and swallowing-related fMRI metrics. Further MRI scans would entail unnecessary radiofrequency exposure and patient travel burden, offering no clinical benefit and contravening the minimal-risk ethical principle.

### 2.9 Outcome measures

#### 2.9.1 Primary outcomes

The Functional Oral Intake Scale (FOIS) is a validated 7-point ordinal scale that assesses functional feeding status, ranging from tube feeding dependence (level 1) to unrestricted oral intake (level 7) (24). It will be used to assess the functional oral intake of PSD patients and as a eligibility criterion for selecting participants. Existing evidence indicates that acupuncture typically improves FOIS scores by 1–2 points (25), though treatment response varies with baseline characteristics, specific acupuncture parameters and intervention duration. For patients with initial scores of 2–4, achieving normal swallowing levels (FOIS ≥ 6) after 4 weeks of acupuncture treatment will be considered indicative of treatment effectiveness (26). The FOIS has been well-established as a reliable outcome measure for tracking longitudinal changes in swallowing function among stroke patients (27). Its standardized design and clinical relevance have led to widespread adoption in dysphagia research.

#### 2.9.2 Secondary outcomes

##### 2.9.2.1 Recovery of the swallowing center and peripheral nerves

(1) fMRI data acquisition and analysis

Functional magnetic resonance imaging (fMRI) will be acquired to measure ReHo and ALFF indicators, which will be assessed before and during treatment. The ReHo indicator quantifies local neural activity synchronization, with changes indicating functional reorganization at the regional level (28). The ALFF metric reflects spontaneous neural activity intensity, thereby indicating the dynamic changes in brain functional reorganization (29).

Structural images are obtained using a TI-3D MPRAGE sequence with a sagittal scan orientation (left-to-right acquisition), yielding high-resolution T1-weighted images of the whole brain. Scan parameters are: repetition time (TR)/echo time (TE) = 2,000 ms/1.97 ms; flip angle (FA) = 8°; number of excitations (NEX) = 0.5; field of view (FOV) = 256 × 256 mm; matrix size = 256 × 256; slice thickness = 1.0 mm; no gap; 192 slices; scan time = 4 min 40 s; total images = 190.

Resting-state fMRI (rs-fMRI) data are acquired using a gradient-echo (GRE) single-shot echo-planar imaging (EPI) sequence. Scan parameters are: TR/TE = 2,000 ms/30 ms; FA = 90°; FOV = 220 × 220 mm; matrix = 64 × 64; slice thickness = 3 mm; gap = 3 mm; 37 slices; 240 time points; scan time = 8 min 8 s; producing 2,037 volumes.

Functional MRI data will undergo a standardized preprocessing pipeline. First, DICOM images will be converted to NIfTI format, followed by the exclusion of participants exhibiting excessive head motion—defined as translation exceeding 2 mm or rotation > 2°—or signal artifacts. Subsequent core preprocessing will include realignment for motion correction, slice timing adjustment, spatial normalization to the MNI152 template at 2 mm^3^ isotropic resolution, nuisance regression incorporating six head motion parameters along with cerebrospinal fluid and white matter signals, and band-pass filtering between 0.01 and 0.08 Hz. Finally, spatial smoothing will be applied using a 6 mm full-width-at-half-maximum Gaussian kernel.

Regions of interest (ROIs) will be defined based on the Automated Anatomical Labeling (AAL) atlas (30). In accordance with previous neuroimaging studies on swallowing mechanisms (31), the ROIs will include the precentral gyrus, insula, and anterior cingulate cortex.

(2) Surface electromyography (sEMG) detection

**FIGURE 3:**
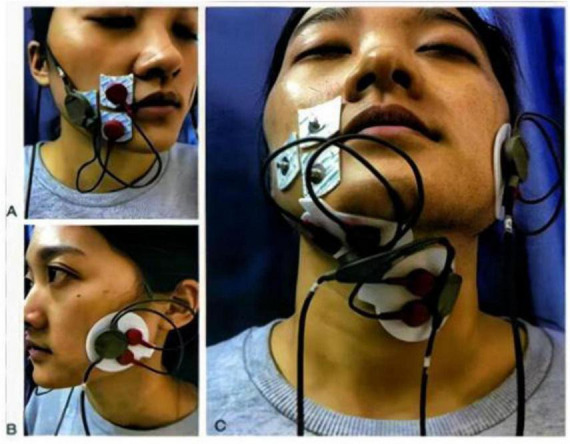
Surface electrode placement locations. **(A)** Right orbicularis oris. **(B)** Left masseter. **(C)** Right submental muscle group; Left infrahyoid muscle group (31).

Surface electromyography (sEMG) testing is used to assess the coordination of local swallowing muscle groups, with evaluations conducted before, during, and after treatment. The tested muscle groups include the upper and lower oral sphincter muscles, masticatory muscles, submental muscle groups (comprising the anterior belly of the digastric muscle, mylohyoid muscle, and geniohyoid muscle), and subhyoid muscle groups (including the thyrohyoid muscle and sternohyoid muscle). The electrode placement locations are illustrated in Figure 3. Primary analysis indicators consist of swallowing action duration (seconds), amplitude of electromyographic activity (mean value), waveform patterns, and swallow count during continuous swallowing testing (32).

##### 2.9.2.2 Quality of living: Swallowing *of Life Questionnaire*

Patients complete the Quality questionnaire under physician guidance after fully understanding each question’s format. The SWAL-QOL (33) comprises 44 items organized into 10 dimensions (totaling 30 items): general burden, appetite, mealtime duration, food selection, communication, fear, mental health, social functioning, fatigue, and sleep, plus a separate 14-item symptom frequency assessment scale (34). Administering the SWAL-QOL before and after treatment provides clear, intuitive measurement of quality-of-life improvements, with higher scores indicating better quality of life (35).

##### 2.9.2.3 Neurological deficit: National *of Health Stroke Scale*

The National InstituteInstitutes of Health Stroke Scale (NIHSS) (36) assesses neurological deficit severity through 15 evaluation items: level of consciousness, gaze, visual fields, facial palsy, motor function of upper and lower limbs, limb ataxia, sensory function, language, dysarthria, and extinction/inattention (37). The scale ranges from 0 to 42 points, with higher scores indicating greater stroke severity (38). Trained researchers score each item based strictly on the patient’s initial responses without providing cues or prompts (37).

##### 2.9.2.4 Swallowing safety and efficacy: Fiberoptic Endoscopic Evaluation of Swallowing

FEES serves as a well-established and reliable tool for assessing swallowing impairment and guiding behavioral interventions (39). During the procedure, a clinician passes a flexible fiberoptic endoscope transnasally to directly visualize the structural integrity of the oropharynx and larynx, while dynamically observing key physiological events such as bolus transit, glottic closure, and laryngeal elevation. These observations provide a quantitative basis for grading the severity of swallowing dysfunction. Swallowing safety is evaluated using the Penetration-Aspiration Scale (PAS) (40), whereas swallowing efficacy is measured with the Yale Pharyngeal Residue Severity Rating Scale (YPRSRS) (41). Both instruments offer objective metrics that help minimize subjective assessment bias and support the development of individualized treatment plans.

To detect silent aspiration during Fiberoptic Endoscopic Evaluation of Swallowing (FEES), the endoscope will be maintained in position for an extended observation period of 45 s following each bolus swallow, allowing for the active capture of delayed aspiration events. Even in the absence of immediate aspiration, the presence of subglottic residue—observed as retained material or contrast-stained liquid below the vocal folds—will be documented as indirect evidence of silent aspiration. For precise quantification, the Penetration-Aspiration Scale (PAS) will be applied with specific grading annotations; for instance, “PAS 5 with thin liquids” will be recorded instead of the generic “PAS 5.” Furthermore, the incidence of silent aspiration will be statistically analyzed separately, and its association with the Functional Oral Intake Scale (FOIS) scores will be examined to reduce bias in therapeutic outcome evaluation.

##### 2.9.2.5 Treatment expectation assessment: Treatment Expectation Questionnaire^1^

Treatment expectations will be assessed using the abbreviated Treatment Expectation Questionnaire (TEX-Q) (42), a 15-item instrument comprising six subscales. This measure was adopted due to the lack of validated tools for assessing treatment expectations in the context of stroke rehabilitation within the Chinese population. Higher scores on the TEX-Q indicate stronger expectations toward the assigned intervention. At baseline, participants in both the acupuncture and rehabilitation control groups will complete the treatment-specific version of the questionnaire, while the healthy control group will be exempt from this assessment. This baseline expectation evaluation will be conducted concurrently with other baseline measurements, immediately after enrollment but prior to the initiation of any treatment.

##### 2.9.2.6 Self-evaluation: patient reported outcome

The patient-reported outcome (PRO) is a standardized questionnaire format typically administered at baseline, during treatment, and during post-treatment follow-up.^2^ It primarily assesses patients’ symptom burden, physical function, mental health, and quality of life, serving as key indicators to evaluate treatment impact on swallowing function and quality of life. These subjective measures complement objective clinical assessments such as the NIHSS (43).

#### 2.9.3 Safety assessment

##### 2.9.3.1 Analysis of adverse events

Treatment-related AEs include symptoms such as throat discomfort or pain, coughing, and aspiration pneumonia caused by the trial intervention or acupuncture. Serious adverse events (SAEs) refer to symptoms that result in hospitalization, persistent or significant disability, functional impairment, life-threatening conditions, or death (44). Additionally, there may be treatment-unrelated AEs that occur outside the treatment period (44).

##### 2.9.3.2 Attribution criteria for adverse events

To differentiate between adverse events (AEs) causally related to acupuncture intervention and those potentially attributable to pre-existing post-stroke depression (PSD), attribution will be determined in accordance with the CIOMS guidelines (45) and the Standards for Reporting Adverse Events in Acupuncture Clinical Trials (46). Detailed criteria are provided in Table 3.

**TABLE 3:** Severity grading criteria for adverse events.

Severity grade	Core definition	Diagnostic criteria
Mild	Symptoms are mild and do not affect daily life or trial participation	1. Local symptoms: Such as mild soreness at the acupuncture site, transient throat numbness and distension (duration < 30 min); 2. Functional impact: No restrictions on daily activities; able to complete interventions normally; 3. Management requirements: No special treatment needed; symptoms resolve spontaneously.
Moderate	Symptoms are noticeable and may moderately interfere with daily activities or trial procedures; they are amenable to symptomatic treatment.	1. Local symptoms: Such as persistent throat discomfort for 1–2 h after acupuncture, or local skin erythema with a diameter of 2–3 cm; 2. Functional impact: Need to adjust daily activities (e.g., reduce intake of hard foods) but without affecting intervention implementation; 3. Management requirements: Symptomatic treatment required (e.g., lozenges for soothing the throat) and symptoms are alleviable.
Severe	Symptoms are severe and significantly affect daily life or result in inability to continue the trial	1. Local symptoms: Such as severe laryngospasm (duration > 10 min) after acupuncture, or skin redness and swelling with a diameter > 3 cm accompanied by pain; 2. Functional impact: Inability to perform daily activities such as eating and swallowing, or require temporary discontinuation of acupuncture or rehabilitation sessions; 3. Management requirements: Medical intervention required (e.g., oxygen therapy, medication); symptoms alleviate slowly.

##### 2.9.3.3 Management of adverse events

**FIGURE 4:**
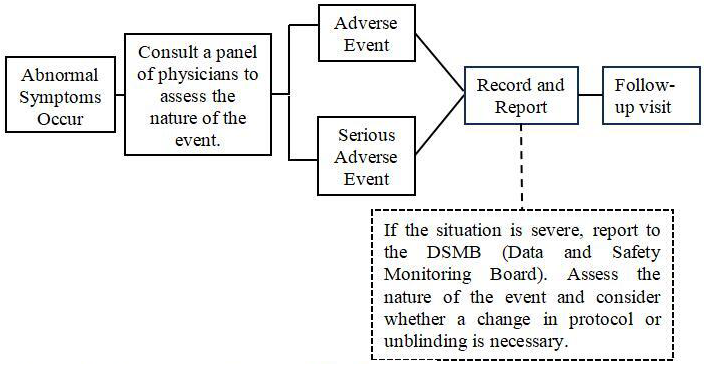
Adverse event management flowchart.

When subjects experience significant AEs, clinicians must document all details and implement appropriate interventions. For SAEs, clinicians should administer emergency measures until the patient’s condition stabilizes. If abnormal laboratory parameters are observed, continuous monitoring should be performed until values normalize, with consideration given to discontinuing observation. Following each acupuncture session, clinicians will document any adverse events (AEs) using an Immediate AE Record Form. The recorded information will include AE symptomatology, time of onset, duration, severity, temporal relationship to the intervention (intra- or post-intervention), association with swallowing actions, and preliminary attribution level. Three days after SAE management, the subject’s general condition should be reassessed with relevant laboratory tests as needed to ensure no impact on subsequent treatment processes and outcomes. Subjects who develop abnormal post-treatment values after having normal pre-treatment values should receive regular follow-up examinations until the follow-up period concludes (Figure 4).

##### 2.9.3.4 Safety analysis

Adverse events will be analyzed using standardized evaluation scales. An independent Safety Committee (SC) will be established, composed of five members with no conflicts of interest: a principal investigator, an acupuncture clinician, a rehabilitation medicine specialist, a statistician, and a patient representative from the stroke rehabilitation community. The SC will be responsible for reviewing and evaluating all safety data and adverse events collected during the trial. Its specific duties will include assessing cumulative serious adverse event (SAE) reports, verifying entries in the Immediate AE Record Form within 24 h, conducting routine reviews every 2 months, and performing expedited reviews of any SAE within 48 h to ensure trial integrity and protect participants’ rights and health. For statistical analysis, Fisher’s exact test will be employed for categorical data comparisons, while the Kruskal-Wallis H test will be utilized for analyzing ordinal categorical variables.

##### 2.9.3.5 Scope of adverse event reporting

In accordance with the CIOMS Guidelines for Adverse Event Reporting (45), this study will comprehensively collect and report all adverse events (AEs). The reporting will include the frequency, severity, and potential relationship to the intervention for every documented AE, thereby ensuring a complete representation of participant safety exposure throughout the trial period.

### 2.10 Evaluation schedule

FOIS, NIHSS, SWAL-QOL, PAS, and YPR-SRS will be assessed at baseline (pre-treatment), 15 and 30 days after treatment initiation, and 30 and 60 days post-treatment completion. Additionally, fMRI-derived ReHo and ALFF metrics, sEMG evaluations, and PRO scores will be obtained at baseline and at 15 and 30 days following treatment initiation. The study timeline for enrollment, intervention, and assessment is detailed in Table 4. All dropouts and their respective reasons will be systematically documented in the case report forms (CRFs).

**TABLE 4:** Study assessment schedule.

Times outcomes	Before treatment	Treatment				Follow-up	
	Day 3–0	Week 1–2	(Day 15)	Week 2–4	(Day 30)	Day 60	Day 90
**Basic medical history collection**							
Informed consent	×						
Basic information	×						
Medical and previous treatment	×						
Complications and its treatment	×						
Location of cerebral infarction	×						
Inclusion/exclusion	×						
Randomization	×						
**Interventions**							
Acupuncture		×		×			
Swallowing rehabilitation training		×		×			
**Assessment**							
FOIS	×		×		×	×	×
ReHo	×		×		×		
ALFF	×		×		×		
sEMG	×		×		×		
PAS	×		×		×	×	×
YPR-SRS	×		×		×	×	×
SWAL-QOL	×		×		×	×	×
NHISS	×		×		×	×	×
PRO	×		×		×		
TEX-Q	×						
**Adverse events observation**							
AE	×^→^						

### 2.11 Data management and quality control

All original clinical data and symptom reports will be systematically recorded in the CRFs and meticulously reviewed by researchers to ensure the accuracy of key study data. All rating scales will be independently administered by two trained assessors blinded to treatment allocation, who will access only baseline information through the medical record system to minimize bias. Clinical practitioners will receive standardized training covering needling localization, depth, angle, and manipulation techniques. To standardize objective measures, FEES procedures will be video-recorded in full with fixed observation durations. fMRI data will be uniformly processed using DPABI V5.1 with pre-specified parameters, while sEMG signals will be automatically recorded without assessor intervention. Subjective outcomes (SWAL-QOL, PRO) will be evaluated by third-party raters separate from the treatment team, with all interviews audio-recorded for verification.

The standardized treatment protocol, developed by the lead neurology department, will be strictly implemented across all participating centers. Any deviations must be reported through an online Protocol Deviation Form within 24 h. Medication adherence will be assessed by pill count at each visit, with adherence outside the 80–120% range classified as non-adherence. Source documents and blood pressure records will be uploaded to the central IWRS for independent quality control monitoring. All protocol deviations will be recorded in real time and incorporated into sensitivity analyses. Before study initiation, all clinical staff, safety assessors, and Safety Committee members will undergo standardized training on adverse event attribution criteria. An independent Data Monitoring Committee (DMC), composed of clinical experts from various departments with no conflicts of interest, will be appointed by the hospital to oversee trial progress and perform regular CRF reviews.

### 2.12 Statistical analysis

#### 2.12.1 Functional imaging evaluation indicators

Statistical analysis is performed using the DPABI V5.1^3^ software package. Whole-Brain Analysis: Paired *t*-tests will be used to compare pre- and post-intervention ALFF, fALFF, ReHo, and FC values within each group (acupuncture and rehabilitation training), as well as between groups after intervention. Based on prior evidence indicating direction-specific neuromodulation by acupuncture in swallowing networks (47, 48), the resulting statistical maps will be corrected using one-tailed Gaussian random field theory (voxel *p* < 0.01, cluster *p* < 0.05). ROI Analysis: Mean ReHo and ALFF values will be extracted from each ROI and analyzed using repeated-measures ANOVA to assess within-group changes over time and between-group differences.

#### 2.12.2 Other indicators and safety assessment indicators

Statistical analysis is performed using SPSS V25.0 software. Continuous variables that conformed to a normal distribution or approximate normal distribution are expressed as mean ± standard deviation ($\bar{x}\,s$), while those that do not conform to a normal distribution are expressed as median and interquartile range [M (P25, P75)]. Categorical variables are described using frequency or percentage. Comparisons between groups of unordered categorical variables are performed using the chi-square test. The incidence of silent aspiration will be compared between groups using Fisher’s exact test. Additionally, PAS scores ≥ 5 will be analyzed as ordinal variables using the Mann-Whitney U test.

#### 2.12.3 Analysis adjusting for treatment expectations

For the primary efficacy outcome (FOIS) and key secondary subjective measures (SWAL-QOL, PRO), analysis of covariance (ANCOVA) or mixed-effects models will be employed, with the total baseline treatment expectation score included as a covariate to control for its potential influence on treatment outcomes.

#### 2.12.4 Analysis of Deqi

Group comparisons will be performed based on the binary classification of “adequate” versus “inadequate” deqi, with the Deqi Quality Index (DQI) included as a covariate in an analysis of covariance (ANCOVA) to assess therapeutic effects. Additionally, a linear mixed-effects model will be employed to examine the significance of the DQI × time interaction.

#### 2.12.5 Statistical control for confounding factors

Potential confounders, including age, sex, PSD duration, baseline NIHSS and FOIS scores, stroke subtype, comorbidities (hypertension, diabetes), total treatment expectation scores, and the Deqi Index (DQI), will be accounted for in the analyses. For primary and secondary efficacy outcomes, repeated-measures mixed-effects models or multiple linear regression will be employed. Stroke subtypes will be examined using stratified analysis, while comorbidities will be assessed via sensitivity analyses.

#### 2.12.6 Predefined subgroup analysis

Subgroup analyses will be prespecified in accordance with subgroup data reporting guidelines (49), with details provided in Table 5 (50–54). The results, which will be Bonferroni-corrected for multiple comparisons, will be reported as secondary exploratory outcomes. Treatment effect differences between the acupuncture and rehabilitation control groups across subgroups will be presented using forest plots.

**TABLE 5:** Predefined subgroup categories and rationale.

Subgroup category	Stratification basis (determined at baseline)	Theoretical rationale
Baseline FOIS score subgroup	Low-level group (FOIS = 2), Medium-level group (FOIS = 3–4)	The severity of baseline swallowing function may affect intervention response. Patients with FOIS = 2 and those with FOIS = 3–4 have different therapeutic goals (e.g., prioritizing “oral feeding safety” for FOIS = 2 vs. “feeding independence” for FOIS = 3–4) (50).
Stroke subtype subgroup	Cortical infarction group, subcortical infarction group	Different infarction locations cause varying degrees of damage to the swallowing neural circuit: cortical infarction primarily impairs the cortical swallowing center, while subcortical infarction affects the cortico-brainstem pathway, which may lead to differences in the regulatory effect of acupuncture (51).
Age subgroup	Young group (18–60 years), Elderly group ( > 60 years)	Elderly patients often exhibit atrophy of swallowing muscles. This anatomical change may result in a slower response to the “neuroregulatory effect” of acupuncture compared to young patients (52).
Baseline NIHSS score subgroup	Mild neurological deficit group (NIHSS ≤ 8), moderate neurological deficit group (NIHSS = 9–16)	Stroke severity is correlated with the prognosis of dysphagia. Patients with moderate neurological deficits may require a higher intensity of intervention to achieve meaningful improvements in swallowing function (53).
Deqi adequacy subgroup (acupuncture group only)	Adequate Deqi group (DQI ≥ 4 and ≥ 2 typical sensations with score ≥ 3), inadequate Deqi group (participants who fail to meet the above criteria)	Deqi is a key link in the efficacy of acupuncture. Adequate Deqi (characterized by typical sensations such as soreness, numbness, distension, and heaviness) may enhance the activation of the swallowing neural circuit, thereby strengthening the therapeutic effect of acupuncture (54).

## 3 Discussion

As a traditional Chinese medical therapy, acupuncture has gained substantial attention for its therapeutic efficacy and mechanistic potential in the rehabilitation of post-stroke dysphagia, with a well-established theoretical foundation. Current evidence from published studies (55–58) demonstrates promising clinical outcomes associated with acupuncture therapy. However, comparative studies evaluating acupuncture versus conventional swallowing rehabilitation training remain limited in both quantity and methodological rigor. Notably, swallowing rehabilitation training represents the internationally recognized gold-standard treatment for post-stroke dysphagia and has been extensively implemented in clinical practice worldwide. To more precisely elucidate the comparative efficacy between acupuncture and standard rehabilitation therapy, future clinical investigations should incorporate rigorous methodological designs, including standardized outcome measures, robust data collection and analytical approaches, and quantitative characterization of acupuncture parameters, thereby enhancing the overall quality and reliability of research findings.

The acupoint selection in this study—comprising bilateral Fengchi (GB20), Wangu (GB12), Yifeng (TE17), and Lianquan (CV23)—was based on classical acupuncture theory and contemporary clinical evidence supporting their efficacy in dysphagia management. These acupoints are recognized for regulating “Tong Guan Li Qiao” (orifice-opening functions). Neuroanatomically, Fengchi (GB20) is located in the posterior cervical triangle, where the occipital artery and vein anastomose with their branches, and the greater occipital nerve emerges. Deep needling toward the pharynx stimulates the vagus (CN X), glossopharyngeal (CN IX), facial (CN VII), and hypoglossal (CN XII) nerves, enhancing vertebrobasilar arterial circulation (59). Wangu (GB12) and Yifeng (TE17) demonstrate particular clinical relevance for post-stroke dysphagia, with controlled trials confirming that deep needling improves swallowing function in pseudobulbar palsy (60). Anatomically, Wangu (GB12) resides in the depression posterior-inferior to the mastoid process, adjacent to glossopharyngeal (IX), accessory (XI), vagus (X), and hypoglossal (XII) nerve pathways, potentially facilitating sensory afferent transmission to the CNS. Yifeng (TE17) lies near the glossopharyngeal (IX), hypoglossal (XII), and vagus (X) nerves, enabling direct modulation of peripheral neural activity to influence upper motor neuron pathways. Lianquan (CV23) is positioned between the thyroid cartilage and hyoid bone, with the epiglottis deep to this region and the laryngeal inlet inferiorly. This area is innervated by vagus (X) and glossopharyngeal (IX) nerve terminals; acupuncture here may activate swallowing musculature and vagal nerve endings, promoting muscle contraction and enhancing brainstem-mediated swallowing reflexes to regulate deglutition (59).

This study incorporates significant methodological improvements over previous research, most notably through the inclusion of a healthy control group to establish baseline physiological parameters, enabling stronger mechanistic investigations and enhanced pathological characterization. By conducting the study at two centers while minimizing multi-center coordination challenges—including inter-site variability in equipment specifications, intervention protocols, and staff training standardization—we enhance methodological rigor. Despite the open-label design, this study incorporates multiple methodological safeguards to mitigate potential biases. Standardized assessments, third-party evaluators, and multimodal verification confined detection bias to an acceptable range. Performance bias was controlled by measuring baseline expectations with standardized scales and including these scores as covariates in efficacy analyses. Pre-specified subgroup analyses and thorough control of confounders minimized false-positive risks from data dredging while supporting evidence-based individualized care.

Our comprehensive efficacy evaluation employs multiple metrics: the Functional Oral Intake Scale (FOIS), fMRI-derived measures ReHo and ALFF, surface electromyography (sEMG), the Swallowing Disorders-Specific Quality of Life Questionnaire (SWAL-QOL), the National Institutes of Health Stroke Scale (NIHSS), Fiberoptic Endoscopic Evaluation of Swallowing (FEES) integrated with the Penetration-Aspiration Scale (PAS) and Yale Pharyngeal Residue Severity Rating Scale (YPR-SRS), and Patient-Reported Outcomes (PRO). Functional MRI (fMRI) serves as a superior non-invasive neuroimaging modality for dynamic neural activity monitoring (61), offering exceptional spatial resolution for mapping cerebral activation patterns across cortical and subcortical structures (thalamus, basal ganglia, cerebellum, and brainstem) (62). Crucially, fMRI captures data under near-physiological conditions, enabling investigation of spontaneous neuronal activity, functional connectivity networks, and neuroplastic changes (63). Previous research demonstrates an inverse correlation between ReHo values in swallowing-related brain regions and dysphagia severity in stroke patients (47), suggesting ReHo alterations may track neural compensation during swallowing recovery (48). Similarly, ALFF—quantifying spontaneous neural activity intensity (64)—shows treatment responsiveness (65). The SWAL-QOL is a validated assessment tool characterized by simplicity of administration, time efficiency, high reliability, and good validity (34). It provides quantitative measurement of swallowing-related quality of life, enabling clinicians to better evaluate patients’ swallowing function and dysphagia severity. The NIHSS employs standardized scoring criteria that ensure inter-rater reliability and comparability (48). Numerous studies have established the NIHSS as a simple yet effective predictive measure for neurological deficits (66–68), which can be used to forecast long-term functional recovery and guide rehabilitation planning. The objective observations from FEES are translated into quantifiable efficacy data using the Penetration-Aspiration Scale (PAS) and Yale Pharyngeal Residue Severity Rating Scale (YPR-SRS), thereby enhancing the precision of therapeutic outcome evaluation for the intervention. This study comprehensively evaluates acupuncture efficacy through four key dimensions: physiological function, cerebral activity, quality of life, and disease severity, thereby generating high-quality, internationally recognized evidence-based findings.

This study has several limitations that warrant consideration. First, the relatively small sample size may reduce statistical power, potentially limiting the generalizability of findings. Additionally, significant heterogeneity in intervention protocols made effective blinding challenging, increasing risks of performance and detection bias. Participant expectations and subjective perceptions may influence self-reported outcomes, potentially compromising the reliability of patient-reported measures. While objective clinical assessments provide valuable data, they remain susceptible to investigator interpretation bias. The follow-up in this trial will conclude at 60 days post-treatment to assess short-term efficacy. Should the primary outcomes prove positive, the research team will seek extended funding after study completion to conduct additional follow-ups at 6 and 12 months. These extended assessments will evaluate swallowing function, pneumonia incidence, and quality of life to determine the long-term durability of treatment effects.

## Footnotes

1
https://osf.io/dr3gc/

2
https://www.healthmeasures.net/index.php?option=com_instruments&view=measure&id=780&Itemid=992

3
http://rfmri.org/dpabi
